# Electronic Package Leaflets for Vaccines: What Are People’s Perceptions in Italy?

**DOI:** 10.3390/vaccines10071075

**Published:** 2022-07-04

**Authors:** Angela Bechini, Fabrizio Chiesi, Barbara Giammarco, Eleonora Gori, Mariarosaria Di Tommaso, Noemi Strambi, Elisabetta Alti, Paola Picciolli, Giovanna Mereu, Maria Grazia Mori, Giovanni Vitali Rosati, Pierre Van Damme, Martina Bamberger, Paolo Bonanni, Sara Boccalini

**Affiliations:** 1 Department of Health Sciences, University of Florence, 50134 Florence, Italy; mariarosaria.ditommaso@unifi.it (M.D.T.); noemi.strambi@unifi.it (N.S.); paolo.bonanni@unifi.it (P.B.); sara.boccalini@unifi.it (S.B.); 2 Central Tuscany Local Health Unit (ASL Toscana-Centro), 50122 Florence, Italy; fabrizio.chiesi@gmail.com (F.C.); elisabetta.alti@uslcentro.toscana.it (E.A.); paola.picciolli@uslcentro.toscana.it (P.P.); giovanna.mereu@uslcentro.toscana.it (G.M.); mariagrazia.mori@uslcentro.toscana.it (M.G.M.); 3 South-East Tuscany Local Health Unit, 58100 Grosseto, Italy; barbara.giammarco@uslsudest.toscana.it; 4 Medical Specialization School of Hygiene and Preventive Medicine, University of Florence, 50134 Florence, Italy; eleonora.gori@unifi.it; 5 FIMP (Italian Paediatrician Federation), 00185 Rome, Italy; giovannivitalirosati@gmail.com; 6 Centre for the Evaluation of Vaccination, Vaccine and Infectious Disease Institute, University of Antwerp, 2000 Antwerp, Belgium; pierre.vandamme@uantwerp.be (P.V.D.); martina.bamberger@uantwerpen.be (M.B.)

**Keywords:** vaccine, electronic leaflet, paper package insert, acceptability, elderly, pregnant women, young parents

## Abstract

In Italy, the paper package leaflet (PPL) is the official document that is approved by the Italian Medicines Agency (AIFA) for each medicine. PPLs of all medicines, including vaccines, are freely available online by accessing the AIFA website. To investigate people’s attitudes toward possible access to the PPLs of vaccines and the acceptability of switching to an electronic package leaflet (e-leaflet) in the future, we surveyed three target groups (pregnant women, young parents, and older people) in Italy, via an online survey. We collected 321 questionnaires from the cohorts, which comprised 104 pregnant women, 105 young parents, and 112 older people. The results indicate in all target groups that health care professionals (HCPs) do not usually offer the vaccine PPL during the vaccination session: only about 10.7% of respondents receive the PPL without asking for it, with pregnant women receiving it the most frequently. The acceptance rate for switching from a PPL to an e-leaflet is fairly high in all target groups (76.9% in pregnant women, 81.9% in young parents, and 66.1% in the elderly), especially if the option exists to request a paper print, to make sure that people with a low level of digital skills can access the PPL information as well. HCPs have an important role in ensuring access to the PPLs of vaccines. HCPs should be trained to inform their patients about the different options for accessing the PPLs (as well as online access) to increase their patients’ knowledge and satisfaction.

## 1. Introduction

In Europe, drug legislation specifies that medicines released into circulation in the European Community must be accompanied by a leaflet containing information that is understandable to patients, explaining the appropriate and safe use of the medicine [1,2]. Vaccines are biological medicines and so they are not exempt from this obligation. The paper package leaflet (PPL) is a document that contains all the information necessary for the correct, appropriate, and safe use of the medicine by patients. The information contained therein is in line with that contained in the summary of product characteristics (SmPC). These documents represent the list of characteristics of the drug, as documented in the chemical/pharmaceutical, preclinical and clinical dossier presented by the pharmaceutical companies to the competent authorities for the purposes of marketing authorization. In Italy, the PPL is therefore an official document approved by the Italian Medicines Agency (AIFA), the content of which is periodically updated. PPLs must contain information that is easily legible, clearly understandable, and indelible [1]. Unfortunately, despite the existence of the European official guidelines on the readability of the leaflet [3], the content of PPLs is not always clear or useful to the recipients [4,5,6]. The PPLs are aimed at a heterogeneous public with extremely varied levels of schooling and education, and the language is not always adjusted to meet the non-specialist’s level of knowledge [7]. Overall, the PPLs could present various readability problems [8]. Information in the PPLs should be given in a schematic and concise way, and language barriers should not prevent people from understanding it [9]. Over the last few years, in accordance with European legislation [10], a simplification of the PPL has been launched. The launched directives establish a series of very specific criteria to facilitate the legibility and understanding of the leaflet by the patient. In response to this legislation and to inform the growing number of people who use the Internet to obtain information about the use and the safety of drugs, particularly vaccines [11], the AIFA made the PPLs available to the population in a digital format, through a medicines database [12]. This database allows the consultation of the SmPCs and PPLs for all drugs authorized in Italy. All published documents are checked and approved by the AIFA and the European Medicines Agency (EMA). The database, which is constantly updated and enriched with new information, is easily accessible and is designed for quick and intuitive consultation, even from smartphones and tablets, thanks to a free application and the QR code system present on the packages of medicines. In addition, the database provides for the possibility of consulting the PPLs in multilingual and audio modes, satisfying the needs of diverse population groups, such as foreigners residing in Italy, the visually impaired community, and people with physical disabilities that prevent them from handling the traditional leaflet printed on paper. Last but not least, the PPLs have an unavoidable environmental footprint because many leaflets are printed every year, demanding huge paper consumption, and the pharmaceutical packaging, including vaccine packaging, must be large enough to contain this paper inside.

In this context, the “Package leaflet project” has been developed, and a collaboration between the University of Antwerp and the University of Florence was established in 2020 [13,14]. The e-leaflet represents an advanced electronic version of the PPL contained in the packaging of medicines and creates a direct channel between the pharmaceutical companies and consumers, allowing them to send and receive information in a simple, fast, and convenient form. The e-leaflet is updated in real time and is always accessible with a free application, offering additional functions, audio contributions, images, videos, help forms for calculating the dosage, and telephone numbers to contact. There is also a document for each language and formulation. It is important to underline that the content of e-leaflets follows the same path of approval as the paper sheets; they are prepared by the pharmaceutical companies and are then approved by the EMA in Europe and by the AIFA in Italy. The “Package leaflet project”, therefore, sets itself the objective of replacing the PPLs of the vaccines with these e-leaflets, at a European level.

The aim of the study is to investigate people’s attitudes toward the possible access and use of the PPLs of vaccines and the acceptability of switching to an e-leaflet in the near future, with the help of three target groups (pregnant women, young parents, and older people) in Italy, particularly in the region of Tuscany.

## 2. Materials and Methods

### 2.1. Study Design

The study was carried out via the administration of a survey to diverse population groups and was developed in Europe with the active participation of four countries (Belgium, France, Italy, and Bulgaria). In Italy, three different target groups were selected and were reached with the help of the Gynecology and Midwifery Department of Careggi University Hospital (Florence), the vaccination clinics, and the pediatricians’ and general practitioners’ clinics of the Central Tuscany local health unit (CT-LHU). The goal was to collect at least 100 replies from each group, with a total of at least 300 entries. The target groups were pregnant women, the parents of young children, and older people. For pregnant women, their progress in pregnancy was not considered. For the parents, we included mothers or fathers of children younger than 12 years old, who were present during the vaccination of their child in the previous two years. For elderly respondents, we included people who were equal to or older than 60 years; we did not make any gender discrimination. We included all the subjects who voluntarily joined the project, read the informative sheet, and signed the informed consent and the processing of personal data consent forms. We excluded all those subjects who did not cooperate and did not finish the questionnaire.

### 2.2. Structure of the Questionnaire

**Figure 1:**
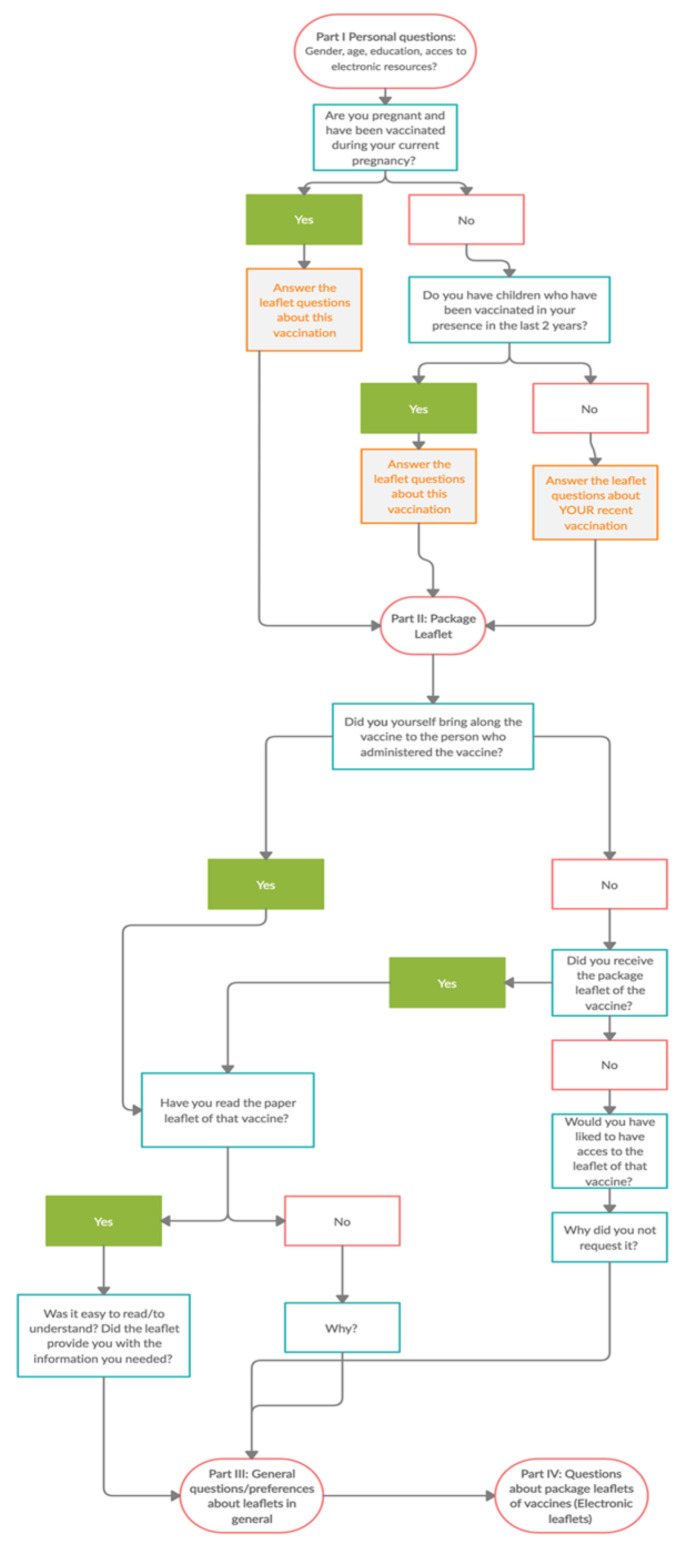
Flow chart of the questionnaire.

The questionnaire, designed by the Belgian developers of the project, contained YES/NO questions, open questions, and multiple-choice questions, with a total number of 30. The time for completion was determined to be about 10 min. The questionnaire consisted of four sections: demographic questions (gender, age, education, access to electronic resources); package leaflet; general questions/preferences about leaflets in general; questions about the package leaflets of vaccines (electronic leaflets) (Figure 1). The questions were related to the most recent vaccination. An Italian version of the questionnaire was prepared by residents of the Specialization Medical School of Hygiene (University of Florence), providing a translation from the English original version.

### 2.3. Collecting and Analysis of Data

The original protocol of the study was based on the administration of a paper version of the questionnaire, with face-to-face interviews conducted by collaborators of the projects, such as residents of the Specialization Medical School of Hygiene (University of Florence), residents of the General Practitioners Specialization course in Tuscany, and gynecologists and midwives at the Careggi University Hospital. Three different settings were identified: vaccination clinics at the CT-LHU and pediatricians’ clinics for young parents, general practitioners’ clinics at the CT-LHU for older people, and gynecology clinics at the Careggi University Hospital for pregnant women. Due to the emergency of the COVID-19 pandemic, which emerged in the initial phase of the project, it was not possible to contact people face-to-face anymore. Therefore, a switch to an online questionnaire was required. Women were contacted during their visits to the hospital and they were invited to fill in the online version of the questionnaire. Elderly people were the most difficult target to reach in Italy. We found some alternative solutions to increase the adhesion for both the elderly and young parents. Firstly, the questionnaire link and QR code were distributed through VaccinarSinToscana.org website and its related Facebook account. Secondly, we contacted regional voluntary organizations and two national elderly support organizations: “Happy Aging” and “Active Citizenship”. In the digital version of the questionnaire, an information letter and informed consent form were included. Data were recorded in an electronic database and a progressive identification number (ID) was assigned to each subject, in order to guarantee anonymity. Data processing and analysis were conducted by means of IBM SPSS Statistic 25 (IBM Corporation, Armonk, NY 10504, United States of America). A descriptive analysis was performed, and results were presented as a percentage or mean ± standard deviation (SD).

### 2.4. Approval Process

The project was approved by the Ethics Committee of Local Health Unit Tuscany Center (Ref. CEAVC 16714/2020). The study was conducted according to the principles of the Helsinki Declaration.

## 3. Results

The study was carried out over the period from 4 March 2020 to 7 August 2020. We enrolled 321 subjects (104 pregnant women, 105 young parents, and 112 older people).

The first part of the questionnaire investigated the population characteristics of the respondents (Table 1).

In the young parents’ group, 85 respondents were female (81.0%) and 20 were male (19.0%), while of the older people, 47 (42.0%) were female and 65 (58.0%) were male; in total, we recruited 236 females (73.5%) and 85 males (26.5%). The overall average age was 49.2 years; the pregnant women’s average age was 34.8 years, 39.9 years for the young parents, and 71.2 years for the older people. Most of the pregnant women had a university degree or higher (56.7%), 35.6% had graduated from high school, and 7.7% had finished primary school. Most young parents had a university degree or higher (64.8%), 31.4% had graduated from high school, and 3.8% had finished primary school. Of the older respondents, 13.4% did not have any educational qualifications, 16.1% had finished primary school, 44.6% had graduated from high school, and 25.9% had a university degree or higher. In general, 4.7% of subjects did not have any educational qualification, 9.3% finished primary school, 37.4% had graduated from high school, and 48.6% had a university degree or higher. Most of the pregnant women (76.9%), young parents (64.8%), and older people (84.8%) did not have a job or training in the healthcare sector. In general, only 24.3% of subjects had a job or training in the healthcare sector. All the pregnant women and young parents had at least one electronic device, while 10.7% of older people did not have any electronic devices.

The second part of the questionnaire contained questions about the PPL of the vaccine that the patients received at their last vaccination (Table 2).

Most pregnant women (95.2%), young parents (93.3%), and older people (92.8%) did not bring the vaccine directly to the person who administered the vaccine. Nearly 17% of pregnant women, 8.2% of young parents, and 6.8% of older people, who did not bring along the vaccine themselves, received the PPL spontaneously from the person who administered it. Only four respondents (two pregnant women, one young parent, and one older person) who did not receive the PPL of the vaccine spontaneously requested and received it. The majority of pregnant women (57.5%) and older people (65.3%), who did not receive the PPL of the vaccine, would not have liked to have access to it, while 59.6% of young parents would have liked to receive it. Pregnant women did not request the PPL because they trusted the person who injected them (40.0%), they were not interested or they did not need it (7.5%), they have already read it in the past (11.3%), or they did not know they could ask for it (47.5%). Young parents did not request the PPL because they trusted the person who injected them (52.8%), they were not interested or they did not need it (3.4%), they have already read it in the past (5.6%), they did not know that they could ask for it (65.2%), or they did not understand it (1.1%). Finally, older people did not request the PPL because they trusted the person who injected them (67.4%), they were not interested or they did not need it (15.8%), they have already read it in the past (2.1%), they did not know that they could ask for it (26.3%), or they did not understand it (2.1%).

**Figure 2:**
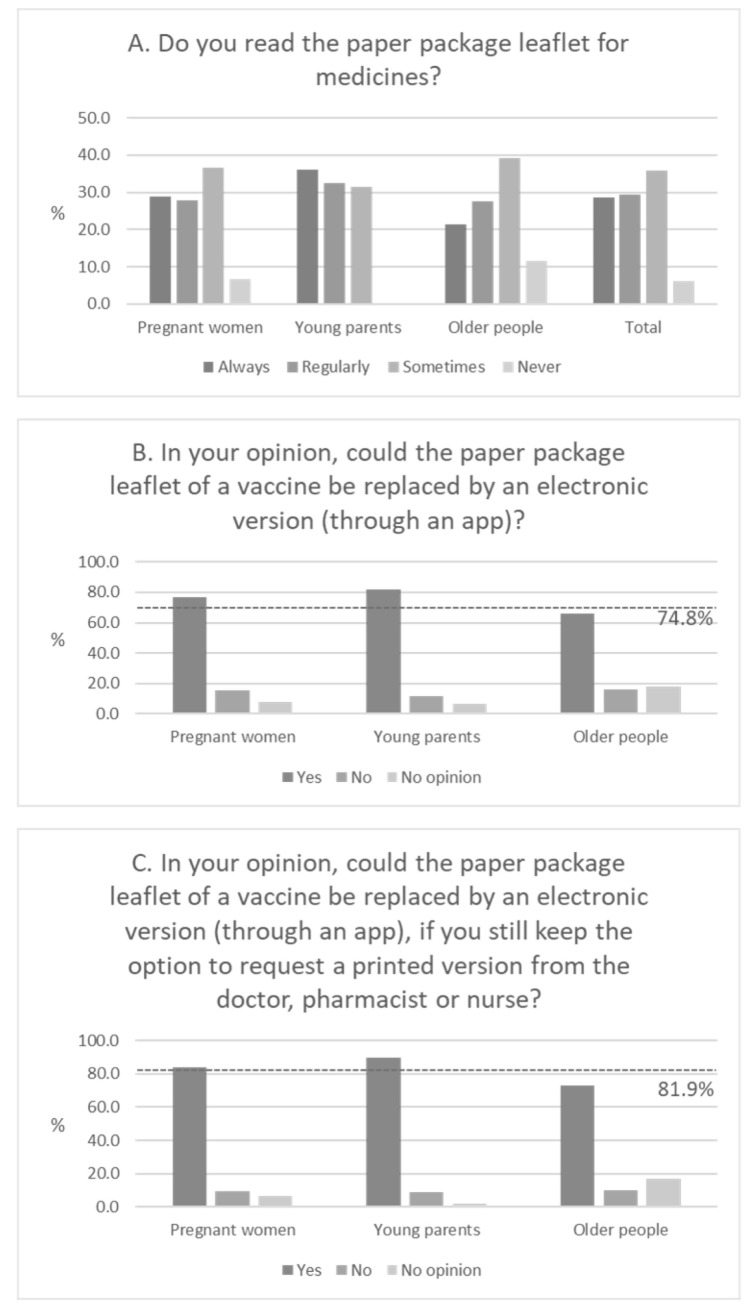
Summary of the main results of the project, with these primary questions: (**A**) Do you read the paper package leaflet for medicines? (**B**) In your opinion, could the paper package leaflet of a vaccine be replaced by an electronic version (available through an app)? (**C**) In your opinion, could the paper package leaflet of a vaccine be replaced by an electronic version (available through an app), if you still have the option to request a printed version from the doctor, pharmacist, or nurse?

The third part of the questionnaire contained questions about PPLs in general (Table 3 and Figure 2).

Many of the subjects claimed to read the PPL sometimes (35.8%), 29.3% read the PPL regularly, and 28.7% always read it; only 6.2% of the subjects claimed never to read the PPL. About 67% of pregnant women and 49.5% of young parents looked on the Internet for the text of the PPL, while only 22.3% of older people used the Internet to search for the PPL. The majority of pregnant women (97.1%), young parents (84.6%), and older people (96.0%), who look on the Internet for the text of PPL know where on the Internet they can find the text of the PPL of medicines and vaccines. Almost 80% of subjects look for this information on Google (the only option given in the questionnaire for a web search engine), 21.1% on specific institutional sites, and 7.5% on a smartphone app, while nobody uses social media to look for information. The majority of pregnant women (58.7%), young parents (79.0%), and older people (72.3%) believed that the PPL of medicines and vaccines is made by the company and is approved by a governmental institution; only 6.9% of the subjects claimed that the PPL of medicines and vaccines is made by the company and is approved by the company.

The fourth part of the questionnaire investigated the opinion of the respondents regarding the possibility of switching to an e-leaflet in the near future (Table 4 and Figure 2).

Most pregnant women (88.5%) and young parents (91.4%) thought that it should be possible to consult the package leaflet of a vaccine electronically, while 23.2% of older people did not have any opinion on that. All pregnant women (100%) and most of the young parents (94.3%) claimed that they were prepared to download a free app to electronically read the package leaflet of a vaccine. Only 58.9% of older people were prepared to download a free app, while almost 30% were not prepared to do so. More than half (53.6%) of the respondents claimed that they would like to receive the information of the package leaflet of vaccines in a video format, while 31.2% of them were not interested in that. The majority of pregnant women (76.9%) and young parents (81.9%) claimed that the PPL of a vaccine could be replaced by an electronic version; for the elderly, 66.1% would be in favor of the replacement of the PPL with an electronic version, 16.1% would be against it, and 17.9% did not have any opinion on the change. The percentages of the respondents in favor of the replacement of the PPL with an electronic version increased for each target group, with 83.7% of the pregnant women, 89.5% of the young parents, and 73.2% of the older people being in favor of the change if the option to request a printed version was maintained.

## 4. Discussion

The aim of this study was to investigate whether Italian people currently have access to and/or read vaccine PPLs and whether they are willing to change to an electronic format in the future. The study also has the purpose of providing indications about the elimination of mandatory PPLs and, at the same time, the introduction of e-leaflets. Particularly, this survey investigated several aspects related to access to the PPL and its use by pregnant women, young parents, and older people. In Italy, vaccines are provided directly by the doctor who administers them; it is unlikely that the vaccine could pick them up at the pharmacy personally. In fact, only a minority of the respondents in our study brought the vaccine themselves to the person who administered it. Acquiring a vaccine and carrying it to the healthcare providers means that people have access to the PPL, which is present in the vaccine box; therefore, only a small minority of pregnant women, young parents, and older people had access to the vaccine PPL before administration. This is not surprising in the case of Italian respondents since, in Italy, vaccines are usually supplied directly by the HCPs during vaccine administration.

Our results indicate in all target groups that the HCPs do not usually offer the vaccine PPL during the vaccination session; in fact, only about 10.7% of respondents received the PPL without asking for it, with pregnant women receiving it the most frequently. It may seem that HCPs are more ready to provide the PPL information spontaneously when pregnant women are involved. The three target groups answered the question of whether they requested and received the PPL from the person who administered the vaccine in the same way. Surprisingly, almost half of the respondents would like to have access to the PPL (mostly young parents) but almost no one in the groups of respondents requested and received the PPL during the vaccination. The most frequent reasons for all target groups of respondents to not request the vaccine PPL were a high level of trust in the HCP and being unaware that they were able to request it. Finally, a high percentage of older people claimed that they were not interested in it or did not need it. Since many respondents think that they cannot ask the HCP for the PPL during the vaccination session, one recommendation for HCPs could be to make the PPL spontaneously available during vaccination. Another suggestion could be to make the PPL available in a different format, to make it more accessible.

Our results indicate that very few respondents never read the PPL for medicines and vaccines; this percentage increased in older people, while all young parents read it at least sometimes but more than one-third read it thoroughly, as confirmed by the literature [15]. This result is in line with the growing willingness of parents to read more information about the many aspects of immunizations, to protect their children [16]. Reading the PPLs has the potential to increase the patients’ knowledge, compliance, and satisfaction [17]. HCPs should be trained and encouraged to educate their patients and inform them about the different options for accessing the PPLs (e.g., online access). Moreover, technical information and PPLs are freely available online by accessing the AIFA website [12]. Pregnant women were the target group that most frequently looked on the Internet for the vaccine PPLs. This result could be related to the growing trend of women searching for information about immunization and vaccination on the Internet [18] and their tendency to pay more attention to their personal health status during pregnancy. Moreover, the Italian Ministry of Health endorsed two documents (in 2018 and in 2019) [19,20], which included recommendations regarding vaccinations for pregnant women. Only 22.3% of the older people used the Internet to search for the vaccine PPLs. The elderly group of respondents was considered to have lower digital literacy and would, therefore, be more critical regarding the use of electronic devices. Younger population groups (pregnant women and young parents), on the other hand, are more likely to use electronic communication, in line with the increasing search for information on medicines and vaccines using the Internet [21]. The majority of people in all three target groups answered that the PPL of medicines and vaccines is made by the company and is approved by a governmental institution. This awareness increases the knowledge and acceptance of vaccines in the general population, particularly among young parents [22].

The great majority of pregnant women and young parents claimed that they would be able to consult the package leaflet of a vaccine electronically and download a free app to read the leaflet electronically. Older people seemed to be less willing to replace the paper with an electronic version. It seems that, when the option to request a printed version exists, more people are willing to accept an electronic format to replace the current paper format. Most interviewees (81.9%) are in favor of introducing an electronic package leaflet, albeit with the guarantee of being able to receive a hard copy at the time of vaccination, if requested.

One limitation of our study could be exemplified by the non-representativeness of the sample, and we did not verify the individual biases of the participants. For example, the average age of pregnant women was older than in the Tuscan study (32.5 years) [23]. Furthermore, the enrolment of participants was difficult because of the national total COVID-19 lockdown. Due to the circumstances of the COVID-19 pandemic, the enrolment times became longer. In general, the acceptance of switching from a paper version to an electronic version is fairly high. Having performed this survey via an online questionnaire, we may have selected a population that was already accustomed to the use of technology. The “use of technology” bias mainly involves the elderly group. Furthermore, in Italy, older people were the most difficult target to reach during our study; we had to find some alternative solutions to increase their adhesion to the survey. We contacted regional voluntary organizations and two national elderly organizations: “Happy Aging” and “Active Citizenship”. On the other hand, having the participants complete the survey electronically provided some advantages: no bias was introduced by the interviewer, and there was no additional, potentially influencing information given during the survey.

Nevertheless, it is important to consider the legibility aspect of a digital version of the leaflet. Indeed, to improve the readability of package leaflets for biological medicines, it is required that organizations promote the understandability and accessibility of this online health information by patients and, thereby, contribute to the appropriate use of medicines and medicine safety [24].

However, the benefits of the e-leaflet are evident for both the consumers and the pharmaceutical companies. On the one hand, the consumers always have more accessible and updated information [21,25]. For example, a mobile app reading a QR code can easily point the user to the correct information about vaccines on a website in text, audio, or video format; in addition, it is easy and quick to update [26]. On the other hand, the pharmaceutical companies can overcome the difficulties in updating PPLs and the restrictions on content imposed by the format required by the regulatory agency, with the addition of clear savings on production costs. The long-term benefits of this replacement could be the greater availability of vaccines throughout the European Union in any language required. Introducing an e-leaflet can offer numerous advantages to the environment, e.g., the use of paper is decreased and the box containing the vaccine can be made smaller, without the PPL needing to make the manufacturing, storage, and distribution more expensive [26].

## 5. Conclusions

The survey shows that the Italian people are generally in favor of accessing the package leaflet information electronically. Furthermore, the acceptance rate for switching from a paper package leaflet to an e-leaflet was fairly high, especially if the option still exists to request a printed version, to make sure that people with poor digital skills can access the PPL information as well.

## Figures and Tables

**Table 1:** Overview of the population characteristics of the respondents, for the three target groups.

	Pregnant Women (*n* = 104)	Young Parents (*n* = 105)	Older People (*n* = 112)	Total (*n* = 321)
**Gender**				
Male	-	20 (19.0%)	65 (58.0%)	85 (26.5%)
Female	104 (100%)	85 (81.0%)	47 (42.0%)	236 (73.5%)
**Age (year)**				
Average age (± SD)	34.8 (± 5.1)	39.9 (± 6.8)	71.2 (± 5.9)	49.2 (± 17.3)
**School education**				
None	0 (0%)	0 (0%)	15 (13.4%)	15 (4.7%)
Primary school	8 (7.7%)	4 (3.8%)	18 (16.1%)	30 (9.3%)
High school	37 (35.6%)	33 (31.4%)	50 (44.6%)	120 (37.4%)
University or higher	59 (56.7%)	68 (64.8%)	29 (25.9%)	156 (48.6%)
**Employment or training in the health care**				
Yes				
No	24 (23.1%)	37 (35.2%)	17 (15.2%)	78 (24.3%)
	80 (76.9%)	68 (64.8%)	95 (84.8%)	243 (75.7%)
**In possession of electronic devices (multiple replies possible)**				
Smartphone	104 (100%)	101 (96.2%)	87 (77.7%)	292 (91.0%)
Tablet	73 (70.2%)	72 (68.6%)	34 (30.4%)	179 (55.8%)
Computer	96 (92.3%)	96 (91.4%)	75 (67.0%)	267 (83.2%)
At least 1 device	100 (100%)	100 (100%)	100 (89.3%)	309 (96.3%)
None	0 (0%)	0 (0%)	12 (10.7%)	12 (3.7%)

**Table 2:** Overview of the answers of the respondents about the vaccine leaflet of the most recent vaccination, for the three target groups.

	Pregnant Women (*n* = 104)	Young Parents (*n* = 105)	Older People (*n* = 112)	Total (*n* = 321)
**Did you bring along the vaccine yourself to the person who administered it?**				
Yes	5 (4.8%)	7 (6.7%)	9 (8.0%)	21 (6.5%)
No	99 (95.2%)	98 (93.3%)	103 (92.0%)	300 (93.5%)
**Did you spontaneously receive the package leaflet of that vaccine from the person who administered it?**				
*Respondents*	99	98	103	300
Yes	17 (17.2%)	8 (8.2%)	7 (6.8%)	32 (10.7%)
No	82 (82.8%)	90 (91.8%)	96 (93.2%)	268 (89.3%)
**Did you request and receive the paper package leaflet of the last vaccine from the person who administered it?**				
*Respondents*	82	90	96	268
Yes	2 (2.4%)	1 (1.1%)	1 (1.0%)	4 (1.5%)
No	80 (97.6%)	89 (88.9%)	95 (99.0%)	264 (98.5%)
**Would you have liked to have access to the paper leaflet of the last vaccine?**				
*Respondents*	80	89	95	264
Yes	34 (42.5%)	53 (59.6%)	33 (34.7%)	120 (45.5%)
No	46 (57.5%)	36 (40.4%)	62 (65.3%)	144 (54.5%)
**Why did you not request it? (Multiple replies possible)**				
*Respondents*	80	89	95	264
Trust	32 (40.0%)	47 (52.8%)	64 (67.4%)	143 (54.2%)
No interest/no need	6 (7.5%)	3 (3.4%)	15 (15.8%)	24 (9.1%)
I have already read it in the past	9 (11.3%)	5 (5.6%)	2 (2.1%)	16 (6.1%)
I did not know I could ask for it	38 (47.5%)	58 (65.2%)	25 (26.3%)	121 (45.8%)
I do not understand it	0 (0%)	1 (1.1%)	2 (2.1%)	3 (1.1%)

**Table 3:** Overview of the answers of the respondents about PPLs in general, for the three groups.

	Pregnant Women (*n* = 104)	Young Parents (*n* = 105)	Older People (*n* = 112)	Total (*n* = 321)
**Do you read the paper package leaflets for medicines?**				
Always	30 (28.8%)	38 (36.2%)	24 (21.4%)	92 (28.7%)
Regularly	29 (27.9%)	34 (32.4%)	31 (27.7%)	94 (29.3%)
Sometimes	38 (36.5%)	33 (31.4%)	44 (39.3%)	115 (35.8%)
Never	7 (6.7%)	0 (0%)	13 (11.6%)	20 (6.2%)
**Do you look on the Internet for the text of the package leaflets of vaccines?**				
Yes	70 (67.3%)	52 (49.5%)	25 (22.3%)	147 (45.8%)
No	34 (32.7%)	53 (50.5%)	87 (77.7%)	174 (54.2%)
**Do you know where on the Internet you can find the text of the package leaflets of medicines and vaccines?**				
*Respondents*	70	52	25	147
Yes	68 (97.1%)	44 (84.6%)	24 (96.0%)	136 (92.5%)
No	2 (2.9%)	8 (5.4%)	1 (4.0%)	11 (7.5%)
**If you look for information about vaccines on the Internet, which statement applies to you? (Multiple replies possible)**				
*Respondents*	70	52	25	147
Google	55 (78.6%)	39 (75.0%)	22 (88.0%)	116 (78.9%)
App	3 (4.3%)	2 (3.8%)	6 (24.0%)	11 (7.5%)
Social media	0 (0%)	0 (0%)	0 (0.0%)	0 (0%)
Specific institutional website	15 (21.4%)	13 (25.0%)	3 (12.0%)	31 (21.1%)
**Which of these two statements is true?**				
The paper package leaflet of medicines and vaccines is made by the company and is approved by a governmental institution	61 (58.7%)	83 (79.0%)	81 (72.3%)	225 (70.1%)
The paper package leaflet of medicines and vaccines is made by the company and approved by the company	5 (4.8%)	5 (4.8%)	12 (10.7%)	22 (6.9%)
I don’t know	38 (36.5%)	17 (16.2%)	19 (17.0%)	74 (23.1%)

**Table 4:** Overview of the answers of the respondents about the possibility of switching to an e-leaflet, shown for the three target groups.

	Pregnant Women (*n* = 104)	Young Parents (*n* = 105)	Older People (*n* = 112)	Total (*n* = 321)
**Would it be possible for you to consult the package leaflet of a vaccine electronically?**				
Yes	92 (88.5%)	96 (91.4%)	78 (69.6%)	266 (82.9%)
No	6 (5.8%)	2 (1.9%)	8 (7.1%)	16 (5.0%)
No opinion	6 (5.8%)	7 (6.7%)	26 (23.2%)	39 (12.1%)
**Are you prepared to download a free app to electronically read the package leaflet of a vaccine?**				
Yes	104 (100%)	99 (94.3%)	66 (58.9%)	269 (8.8%)
No	0 (0%)	2 (1.9%)	33 (29.5%)	35 (10.9%)
No opinion	0 (0%)	4 (3.8%)	13 (11.6%)	17 (5.3%)
**Would you like to receive the information from the package leaflet of vaccines in a video format?**				
Yes	59 (56.7%)	61 (58.1%)	52 (46.4%)	172 (53.6%)
No	32 (30.8%)	27 (25.7%)	41 (36.6%)	100 (31.2%)
No opinion	13 (12.5%)	17 (16.2%)	19 (17.0%)	49 (15.3%)
**In your opinion, could the paper package leaflet of a vaccine be replaced by an electronic version (available through an app)?**				
Yes	80 (76.9%)	86 (81.9%)	74 (66.1%)	240 (74.8%)
No	16 (15.4%)	12 (11.4%)	18 (16.1%)	46 (14.3%)
No opinion	8 (7.7%)	7 (6.7%)	20 (17.9%)	35 (10.9%)
**In your opinion, could the paper package leaflet of a vaccine be replaced by an electronic version (available through an app), if you still have the option to request a printed version from the doctor, pharmacist, or nurse?**				
Yes	87 (83.7%)	94 (89.5%)	82 (73.2%)	263 (81.9%)
No	10 (9.6%)	9 (8.6%)	11 (9.8%)	30 (9.3%)
No opinion	7 (6.7%)	2 (1.9%)	19 (17.0%)	28 (8.7%)

## Data Availability

The data presented in this study are available on request from the corresponding author.
